# Supplementary material for: Sequential stacking link prediction algorithms for temporal networks
Source: Nat Commun. 2024 Feb 14;15:1364. doi: 10.1038/s41467-024-45598-0 (PMC10866871; doi:10.1038/s41467-024-45598-0)
Supplement: Supplementary file 1 — Supplementary Information [file 41467_2024_45598_MOESM1_ESM.pdf]

Supplementary Information for Sequential Stacking Link Prediction Algorithms for Temporal Networks

A: Real-World Data

The 19 publicly-available real-world temporal network datasets used in this study are obtained through the Index of Complex Networks (ICON) (1), the Stanford Network Analysis Project (SNAP) (2), and Network Repository (3). The datasets are processed to collect the temporal details into layers, with each layer corresponding to a non-overlapping time window with length based on the structure and time frame in the data — usually years, months, hours, or minutes. We list detailed information about the data and their citations in Table SI1.

Table SI1: Real-world Datasets Information

Dataset Name	Acronym	Nodes	Edges	Layers	References
College Message	collgemsg	1899	59835	7	(4) (2)
Bitcoin Alpha Trust	bitcoin	3783	24186	63	(5) (6) (2)
Kaggle chess players	chess	7301	65053	97	(7) (1)
Bitcoin OTC trust network	obitcoin	5881	35592	63	(5) (6) (2)
Brazilian prostitution network	obrazil	26836	50632	100	(8) (1)
Manufacturing emails	radoslaw	167	82927	9	(9) (1)
Reality mining proximity	mit	96	1,086,404	9	(10) (1)
London bike sharing (2014)	london	750	1469851	24	(11) (1)
Ant Colony1 Network	ant1	113	112000	41	(12) (3)
Ant Colony2 Network	ant2	131	140000	41	(12) (3)
Ant Colony3 Network	ant3	160	241000	41	(12) (3)
Ant Colony4 Network	ant4	102	82000	41	(12) (3)
Ant Colony5 Network	ant5	152	194000	41	(12) (3)
Ant Colony6 Network	ant6	164	247000	39	(12) (3)
Facebook Forum	fbforum	899	34000	6	(3)
Facebook Messages	fbmsg	2000	60000	8	(3)
Email DNC	emaildnc	2000	37000	33	(3)
Biology network	bionet1	2684	20000	37	(13) (3)
Biology network	bionet2	2684	20000	37	(13) (3)

B: Topological Network Features

The topological features used in our sequential feature stacking are listed in Table SI2.

C: Oracle-level AUC

To calculate the theoretically optimal level of link predictability in our synthetic temporal networks, we exploit the mathematical equivalence of the Area Under the ROC Curve (AUC) and the binary classification probability that a prediction algorithm Θ assigns a higher score to a missing link (true positive) than to a non-edge (true negative):

$$AUC = Pr(tes > tnes). \tag{SI1}$$

For the calculation here, we assume all the community assignments in the partition are known to the prediction algorithm Θ. Thus, we will refer to this as the "oracle" level AUC score. We let d_i be the degree of node i, m_r be the number of edges in cluster r, the dependency of each cluster across time is represented as l_r, and the number of possible edges is σ. Using the Eq. SI1, we can use Monte Carlo samples of the generated networks to estimate an upper bound AUC score for the community-label T-SBM. We sample 10,000 pairs of true edges and true non-edges and compute the score of each pair based on the true community labels that an Oracle model can infer from the topological structure in the target layer. Due to the fact that we utilized the true labels, which can only be achieved if we are within the deep detectable range (19), this is an upper bound.

(a) Global Topological Features:

Abbreviation	Description	Reference
N	number of nodes	(14)
OE	number of edges	(14)
AD	average degree	(14)
VD	variance of degree distribution	(14)
ND	network diameter	(14)
DA	degree assortativity of graph	(15)
NT	network transitivity (clustering coefficient)	(14)
ACC	average local clustering coefficient	(14)

(b) Pairwise (Dyadic) Topological Features:

CN	common neighbours of i, j	(15)
SP	shortest path between i, j	(15)
LHN	Leichet-Holme-Newman index of negihbor sets of i, j	(16)
PPR	jth entry of personalized pagerank of node i	(17)
PA	preferential attachment(degree product) of i, j	(15)
JC	Jaccard's coefficient neighbor sets of i, j	(15)
AA	Adameic/Adar index of i, j	(15)
RA	Resource allocation index of i, j	(17)
LRA	entry i, j in low rank approximation (LRA) via singular value decomposition (SVD)	(18)
dLRA	dot products of node i and j in LRA via SVD for each pair of nodes i, j	(18)
mLRA	average of entries of i and j's neighbors in low rank approximation	(18)
LRA-approx	a truncated version of LRA	(18)
dLRA-approx	a truncated version of dLRA	(18)
mLRA-approx	a truncated version of mLRA	(18)

(c) Node-Level Topological Features:

LCC_i, LCC_j	local clustering coefficeints for i and j	(17)
AND_i, AND_j	average neighbor degrees for i and j	(17)
$SPBC_i, SPBC_j$	shortest-path betweenness centralities for i and j	(17)
CC_i, CC_j	closeness centralities for i and j	(17)
DC_i, DC_j	degree centralities for i and j	(17)
EC_i, EC_j	eigenvector centralities for i and j	(17)
KC_i, KC_j	katz centralities for i and j	(17)
LMT_i, LNT_j	local number of triangles for i and j	(17)
PR_i, PR_j	pagerank values for i and j	(17)
LC_i, LC_j	local centralities for i and j	(17)

Table SI2: The three categories of topological features used in our sequential stacking of static features for link prediction: global features, dyadic features, and node-level features.

Now for a single layer model, the AUC score for any SBM model could be calculated by:

$$AUC = Pr(tes > tnes)$$
$$= Pr(tes > tnes|both_{inside})Pr(both_{inside})\sigma$$
$$+ Pr(tes > tnes|both_{outside})Pr(both_{outside})\sigma$$
$$+ Pr(tes > tnes|te_{inside}, tne_{outside})Pr(te_{inside}, tne_{outside})\sigma$$
$$+ Pr(tes > tnes|te_{outside}, tne_{inside})Pr(te_{outside}, tne_{inside})\sigma \, .$$

For the community-label T-SBM, the theoretical predictability can be calculated in the same manner as for the static network. This is because of the assumption we made regarding the community assignment. The only dependence between the previous temporal layer G_t and next layer G_{t+1} is its community label correlation. However, since we have already assumed the community label of G_{t+1} is known to Θ, there is no more information we could gain from the previous temporal layer. Thus, in the case for the community-label T-SBM, we could calculate the oracle AUC score exactly as in Ghasemian et al. (20). Note that the above equation assumes that $\mu = 0$, which assume the edges all lie within communities (21), which thus gives the highest AUC score among all the value of μ since it adds on the randomness in the network. In this setting we then have the following values.

- First term

$$Pr(tes > tnes|both_{inside})$$
$$= \sum_{u_1,v_1,u_2,v_2} \mathbf{1}(u_1 v_1 > u_2 v_2) \frac{p((i_1,j_1) \in E, (i_2,j_2) \notin E || d_{i1,2} = u_{1,2}, d_{j1,2} = v_{1,2})}{p((i_1,j_1) \in E, (i_2,j_2) \notin E)}$$
$$\times p(d_{i1} = u_1) p(d_{j1} = v_1) p(d_{i2} = u_2) p(d_{j2} = v_2)$$

- Second term

$$Pr(tes > tnes|both_{outside})$$
$$= \sum_{u_1,v_1,u_2,v_2} \mathbf{1}(u_1 v_1 > u_2 v_2) \frac{p((i_1,j_1) \in E, (i_2,j_2) \notin E || d_{i1,2} = u_{1,2}, d_{j1,2} = v_{1,2})}{p((i_1,j_1) \in E, (i_2,j_2) \notin E)}$$
$$\times p(d_{i1} = u_1) p(d_{j1} = v_1) p(d_{i2} = u_2) p(d_{j2} = v_2)$$

- Third term

$$Pr(tes > tnes|te_{inside}, tne_{outside})$$
$$= \sum_{u_1,v_1,u_2,v_2} \mathbf{1}(u_1 v_1 m_{rr} > u_2 v_2 m_{rs}) \frac{p((i_1, j_1) \in E, (i_2, j_2) \notin E || d_{i1,2} = u_{1,2}, d_{j1,2} = v_{1,2})}{p((i_1, j_1) \in E, (i_2, j_2) \notin E)}$$
$$\times p(d_{i1} = u_1) p(d_{j1} = v_1) p(d_{i2} = u_2) p(d_{j2} = v_2)$$

- Fourth term

$$Pr(tes > tnes|te_{outside}, tne_{inside})$$
$$= \sum_{u_1,v_1,u_2,v_2} \mathbf{1}(u_1 v_1 m_{rs} > u_2 v_2 m_{rr}) \frac{p((i_1, j_1) \in E, (i_2, j_2) \notin E || d_{i1,2} = u_{1,2}, d_{j1,2} = v_{1,2})}{p((i_1, j_1) \in E, (i_2, j_2) \notin E)}$$
$$\times p(d_{i1} = u_1) p(d_{j1} = v_1) p(d_{i2} = u_2) p(d_{j2} = v_2)$$

For the edge-correlated T-SBM, we modify the equation by adding the fact that the T-SBM now keeps each edge with probability ρ. Under this scenario, we actually only need to modify one point in the previous single-layer calculation. Because again we have full information on the node label, the only thing that is different in this case is that there is also a higher chance that we keep an edge and vice versa. Note that again we have:

We can estimate the oracle AUC score SI1 by Monte Carlo samples of the generated networks to calculate this equation, where we sample 10000 pairs of true edges and true non-edges, and compute the score of these pairs using the following equation from Pamfil et. al (22), where g is the planted partition, $A^{(t)}$ and $A^{(t+1)}$ are the adjacency matrices of the consecutive layers of t and $t+1$, $p^{(t)}$ and $p^{(t+1)}$ are the intralayer edge probability matrices for the same two layers, θ is the degrees of nodes normalized by the mean degrees, and q is the couple edge probability between these consecutive layers that the oracle model is aware of, — detailed discussion about notation and calculation could be found in the section of correlated degree-corrected SBMs and Table 1 in Pamfil et.al (22). Note that we also generalize the notation in Pamfil et.al (22) from two layers case to multiple temporal layers.

$$\mathbb{P}(\mathbf{A}_{ij}^{(t+1)} = 1 | \mathbf{A}_{ij}^{(t)} = 1) = \frac{\sqrt{\theta_i^t \theta_j^t \theta_i^{t+1} \theta_j^{t+1}} q_{rs}}{\theta_i^t \theta_j^t p_{rs}^t} \tag{SI2}$$

$$\mathbb{P}(\mathbf{A}_{ij}^{(t+1)} = 1 | \mathbf{A}_{ij}^{(t)} = 0) = \frac{\theta_i^{t+1} \theta_j^{t+1} p_{rs}^{t+1} - \sqrt{\theta_i^t \theta_j^t \theta_i^{t+1} \theta_j^{t+1}} q_{rs}}{1 - \theta_i^t \theta_j^t p_{rs}^t} \tag{SI3}$$

Also, we can compute the AUC score exactly by expanding this equation for all different configurations as follows. We use the same notation from before, but note here we have E_{t+1} representing the target layer and E_t the layer before.

$$\begin{aligned}
AUC &= Pr(tes > tnes) \\
&= Pr(tes > tnes | both_{inside}, both_{edges}) Pr(both_{inside}, both_{edges}) \sigma \\
&+ Pr(tes > tnes | both_{inside}, both_{non-edges}) Pr(both_{inside}, both_{non-edges}) \sigma \\
&+ Pr(tes > tnes | both_{inside}, te_{edges}\, tne_{non-edges}) Pr(both_{inside}, te_{edges}\, tne_{non-edge}) \sigma \\
&+ Pr(tes > tnes | both_{inside}, te_{non-edges}\, tne_{edges}) Pr(both_{inside}, te_{non-edges}\, tne_{edges}) \sigma \\
&+ Pr(tes > tnes | both_{outside}, both_{edges}) Pr(both_{outside}, both_{edges}) \sigma \\
&+ Pr(tes > tnes | both_{outside}, both_{non-edges}) Pr(both_{outside}, both_{non-edges}) \sigma \\
&+ Pr(tes > tnes | both_{outside}, te_{edges}\, tne_{non-edges}) Pr(both_{outside}, te_{edges}\, tne_{non-edges}) \sigma \\
&+ Pr(tes > tnes | both_{outside}, te_{non-edges}\, tne_{edges}) Pr(both_{outside}, te_{non-edges}\, tne_{edges}) \sigma \\
&+ Pr(tes > tnes | te_{inside}, tne_{outside}, both_{edges}) Pr(te_{inside}, tne_{outside}, both_{edges}) \sigma \\
&+ Pr(tes > tnes | te_{inside}, tne_{outside}, both_{non-edges}) Pr(te_{inside}, tne_{outside}, both_{non-edges}) \sigma \\
&+ Pr(tes > tnes | te_{inside}, tne_{outside}, te_{edges}\, tne_{non-edges}) Pr(te_{inside}, tne_{outside}, te_{edges}\, tne_{non-edges}) \sigma \\
&+ Pr(tes > tnes | te_{inside}, tne_{outside}, te_{non-edges}\, tne_{edges}) Pr(te_{inside}, tne_{outside}, te_{non-edges}\, tne_{edges}) \sigma \\
&+ Pr(tes > tnes | te_{outside}, tne_{inside}, both_{edges}) Pr(te_{outside}, tne_{inside}, both_{edges}) \sigma \\
&+ Pr(tes > tnes | te_{outside}, tne_{inside}, both_{non-edges}) Pr(te_{outside}, tne_{inside}, both_{non-edges}) \sigma \\
&+ Pr(tes > tnes | te_{outside}, tne_{inside}, te_{edges}\, tne_{non-edges}) Pr(te_{outside}, tne_{inside}, te_{edges}\, tne_{non-edges}) \sigma \\
&+ Pr(tes > tnes | te_{outside}, tne_{inside}, te_{non-edges}\, tne_{edges}) Pr(te_{outside}, tne_{inside}, te_{non-edges}\, tne_{edges}) \sigma
\end{aligned}$$

- First term

$$Pr(tes\ >\ tnes|both_{\,inside}, both_{\,edges})$$
$$= \sum_{u_1,v_1,u_2,v_2} \mathbf{1}(u_1v_1 > u_2v_2)$$
$$\frac{p((i_1,j_1) \in E_{T+1}, (i_2,j_2) \notin E_{T+1} | (i_1,j_1) \in E_T, (i_2,j_2) \in E_T, d_{i1,2} = u_{1,2}, d_{j1,2} = v_{1,2})}{p((i_1,j_1) \in E_T, (i_2,j_2) \in E_T, (i_1,j_1) \in E_{T+1}, (i_2,j_2) \notin E_{T+1})}$$
$$\times p(d_{i1} = u_1)p(d_{j1} = v_1)p(d_{i2} = u_2)p(d_{j2} = v_2)$$

- Second term

$$Pr(tes\ >\ tnes|both_{\,inside}, both_{\,non-edges})$$
$$= \sum_{u_1,v_1,u_2,v_2} \mathbf{1}(u_1v_1 > u_2v_2)$$
$$\frac{p((i_1,j_1) \in E_{T+1}, (i_2,j_2) \notin E_{T+1} | (i_1,j_1) \notin E_T, (i_2,j_2) \notin E_T, d_{i1,2} = u_{1,2}, d_{j1,2} = v_{1,2})}{p((i_1,j_1) \notin E_T, (i_2,j_2) \notin E_T, (i_1,j_1) \in E_{T+1}, (i_2,j_2) \notin E_{T+1})}$$
$$\times p(d_{i1} = u_1)p(d_{j1} = v_1)p(d_{i2} = u_2)p(d_{j2} = v_2)$$

- Third term

$$Pr(tes\ >\ tnes|both_{\,inside}, te_{edges}\ tne_{non-edges})$$
$$= \sum_{u_1,v_1,u_2,v_2} \mathbf{1}(u_1v_1l_{rr} > u_2v_2l_{rs})$$
$$\frac{p((i_1,j_1) \in E_{T+1}, (i_2,j_2) \notin E_{T+1} | (i_1,j_1) \in E_T, (i_2,j_2) \notin E_T, d_{i1,2} = u_{1,2}, d_{j1,2} = v_{1,2})}{p((i_1,j_1) \in E_T, (i_2,j_2) \notin E_T, (i_1,j_1) \in E_{T+1}, (i_2,j_2) \notin E_{T+1})}$$
$$\times p(d_{i1} = u_1)p(d_{j1} = v_1)p(d_{i2} = u_2)p(d_{j2} = v_2)$$

- Fourth term

$$Pr(tes\ >\ tnes|both_{\,inside}, te_{non-edges}\ tne_{edges})$$
$$= \sum_{u_1,v_1,u_2,v_2} \mathbf{1}(u_1v_1l_{rs} > u_2v_2l_{rr})$$
$$\frac{p((i_1,j_1) \in E_{T+1}, (i_2,j_2) \notin E_{T+1} | (i_1,j_1) \notin E_T, (i_2,j_2) \in E_T, d_{i1,2} = u_{1,2}, d_{j1,2} = v_{1,2})}{p((i_1,j_1) \notin E_T, (i_2,j_2) \in E_T, (i_1,j_1) \in E_{T+1}, (i_2,j_2) \notin E_{T+1})}$$
$$\times p(d_{i1} = u_1)p(d_{j1} = v_1)p(d_{i2} = u_2)p(d_{j2} = v_2)$$

- Fifth term

$$Pr(tes\ >\ tnes|both_{\,outside}, both_{\,edges})$$
$$= \sum_{u_1,v_1,u_2,v_2} \mathbf{1}(u_1v_1 > u_2v_2)$$
$$\frac{p((i_1,j_1) \in E_{T+1}, (i_2,j_2) \notin E_{T+1} | (i_1,j_1) \in E_T, (i_2,j_2) \in E_T, d_{i1,2} = u_{1,2}, d_{j1,2} = v_{1,2})}{p((i_1,j_1) \in E_T, (i_2,j_2) \in E_T, (i_1,j_1) \in E_{T+1}, (i_2,j_2) \notin E_{T+1})}$$
$$\times p(d_{i1} = u_1)p(d_{j1} = v_1)p(d_{i2} = u_2)p(d_{j2} = v_2)$$

- Sixth term

$$Pr(tes > tnes|both_{\,outside}, both_{\,non-edges})$$
$$= \sum_{u_1,v_1,u_2,v_2} \mathbf{1}(u_1v_1 > u_2v_2)$$
$$\frac{p((i_1,j_1) \in E_{T+1}, (i_2,j_2) \notin E_{T+1}|(i_1,j_1) \notin E_T, (i_2,j_2) \notin E_T, d_{i1,2} = u_{1,2}, d_{j1,2} = v_{1,2})}{p((i_1,j_1) \notin E_T, (i_2,j_2) \notin E_T, (i_1,j_1) \in E_{T+1}, (i_2,j_2) \notin E_{T+1})}$$
$$\times\, p(d_{i1} = u_1)p(d_{j1} = v_1)p(d_{i2} = u_2)p(d_{j2} = v_2)$$

- Seventh term

$$Pr(tes > tnes|both_{\,outside}, te_{edges}\, tne_{non-edges})$$
$$= \sum_{u_1,v_1,u_2,v_2} \mathbf{1}(u_1v_1l_{rr} > u_2v_2l_{rs})$$
$$\frac{p((i_1,j_1) \in E_{T+1}, (i_2,j_2) \notin E_{T+1}|(i_1,j_1) \in E_T, (i_2,j_2) \notin E_T, d_{i1,2} = u_{1,2}, d_{j1,2} = v_{1,2})}{p((i_1,j_1) \in E_T, (i_2,j_2) \notin E_T, (i_1,j_1) \in E_{T+1}, (i_2,j_2) \notin E_{T+1})}$$
$$\times\, p(d_{i1} = u_1)p(d_{j1} = v_1)p(d_{i2} = u_2)p(d_{j2} = v_2)$$

- Eighth term

$$Pr(tes > tnes|both_{\,outside}, te_{non-edges}\, tne_{edges})$$
$$= \sum_{u_1,v_1,u_2,v_2} \mathbf{1}(u_1v_1l_{rs} > u_2v_2l_{rr})$$
$$\frac{p((i_1,j_1) \in E_{T+1}, (i_2,j_2) \notin E_{T+1}|(i_1,j_1) \notin E_T, (i_2,j_2) \in E_T, d_{i1,2} = u_{1,2}, d_{j1,2} = v_{1,2})}{p((i_1,j_1) \notin E_T, (i_2,j_2) \in E_T, (i_1,j_1) \in E_{T+1}, (i_2,j_2) \notin E_{T+1})}$$
$$\times\, p(d_{i1} = u_1)p(d_{j1} = v_1)p(d_{i2} = u_2)p(d_{j2} = v_2)$$

- Ninth term

$$Pr(tes > tnes|te_{\,inside}, tne_{\,outside}, both_{\,edges})$$
$$= \sum_{u_1,v_1,u_2,v_2} \mathbf{1}(u_1v_1m_{rr} > u_2v_2m_{rs})$$
$$\frac{p((i_1,j_1) \in E_{T+1}, (i_2,j_2) \notin E_{T+1}|(i_1,j_1) \in E_T, (i_2,j_2) \in E_T, d_{i1,2} = u_{1,2}, d_{j1,2} = v_{1,2})}{p((i_1,j_1) \in E_T, (i_2,j_2) \in E_T, (i_1,j_1) \in E_{T+1}, (i_2,j_2) \notin E_{T+1})}$$
$$\times\, p(d_{i1} = u_1)p(d_{j1} = v_1)p(d_{i2} = u_2)p(d_{j2} = v_2)$$

- Tenth term

$$Pr(tes > tnes|te_{\,inside}, tne_{\,outside}, both_{\,non-edges})$$
$$= \sum_{u_1,v_1,u_2,v_2} \mathbf{1}(u_1v_1m_{rr} > u_2v_2m_{rs})$$
$$\frac{p((i_1,j_1) \in E_{T+1}, (i_2,j_2) \notin E_{T+1}|(i_1,j_1) \notin E_T, (i_2,j_2) \notin E_T, d_{i1,2} = u_{1,2}, d_{j1,2} = v_{1,2})}{p((i_1,j_1) \notin E_T, (i_2,j_2) \notin E_T, (i_1,j_1) \in E_{T+1}, (i_2,j_2) \notin E_{T+1})}$$
$$\times\, p(d_{i1} = u_1)p(d_{j1} = v_1)p(d_{i2} = u_2)p(d_{j2} = v_2)$$

- Eleventh term

$$Pr(tes > tnes|te_{\,inside}, tne_{\,outside}, te_{edges}\; tne_{non-edges})$$
$$= \sum_{u_1,v_1,u_2,v_2} \mathbf{1}(u_1v_1m_{rr}l_{rr} > u_2v_2m_{rs}l_{rs})$$
$$\frac{p((i_1,j_1) \in E_{T+1}, (i_2,j_2) \notin E_{T+1}|(i_1,j_1) \in E_T, (i_2,j_2) \notin E_T, d_{i1,2} = u_{1,2}, d_{j1,2} = v_{1,2})}{p((i_1,j_1) \in E_T, (i_2,j_2) \notin E_T, (i_1,j_1) \in E_{T+1}, (i_2,j_2) \notin E_{T+1})}$$
$$\times p(d_{i1} = u_1)p(d_{j1} = v_1)p(d_{i2} = u_2)p(d_{j2} = v_2)$$

- Twelfth term

$$Pr(tes > tnes|te_{\,inside}, tne_{\,outside}, te_{non-edges}\; tne_{edges})$$
$$= \sum_{u_1,v_1,u_2,v_2} \mathbf{1}(u_1v_1m_{rr}l_{rs} > u_2v_2m_{rs}l_{rr})$$
$$\frac{p((i_1,j_1) \in E_{T+1}, (i_2,j_2) \notin E_{T+1}|(i_1,j_1) \notin E_T, (i_2,j_2) \in E_T, d_{i1,2} = u_{1,2}, d_{j1,2} = v_{1,2})}{p((i_1,j_1) \notin E_T, (i_2,j_2) \in E_T, (i_1,j_1) \in E_{T+1}, (i_2,j_2) \notin E_{T+1})}$$
$$\times p(d_{i1} = u_1)p(d_{j1} = v_1)p(d_{i2} = u_2)p(d_{j2} = v_2)$$

- Thirteenth term

$$Pr(tes > tnes|te_{\,outside}, tne_{\,inside}, both_{\,edges})$$
$$= \sum_{u_1,v_1,u_2,v_2} \mathbf{1}(u_1v_1m_{rs} > u_2v_2m_{rr})$$
$$\frac{p((i_1,j_1) \in E_{T+1}, (i_2,j_2) \notin E_{T+1}|(i_1,j_1) \in E_T, (i_2,j_2) \in E_T, d_{i1,2} = u_{1,2}, d_{j1,2} = v_{1,2})}{p((i_1,j_1) \in E_T, (i_2,j_2) \in E_T, (i_1,j_1) \in E_{T+1}, (i_2,j_2) \notin E_{T+1})}$$
$$\times p(d_{i1} = u_1)p(d_{j1} = v_1)p(d_{i2} = u_2)p(d_{j2} = v_2)$$

- Fourteenth term

$$Pr(tes > tnes|te_{\,outside}, tne_{\,inside}, both_{\,non-edges})$$
$$= \sum_{u_1,v_1,u_2,v_2} \mathbf{1}(u_1v_1m_{rs} > u_2v_2m_{rr})$$
$$\frac{p((i_1,j_1) \in E_{T+1}, (i_2,j_2) \notin E_{T+1}|(i_1,j_1) \notin E_T, (i_2,j_2) \notin E_T, d_{i1,2} = u_{1,2}, d_{j1,2} = v_{1,2})}{p((i_1,j_1) \notin E_T, (i_2,j_2) \notin E_T, (i_1,j_1) \in E_{T+1}, (i_2,j_2) \notin E_{T+1})}$$
$$\times p(d_{i1} = u_1)p(d_{j1} = v_1)p(d_{i2} = u_2)p(d_{j2} = v_2)$$

- Fifteenth term

$$Pr(tes > tnes|te_{\,outside}, tne_{\,inside}, te_{edges}\; tne_{non-edges})$$
$$= \sum_{u_1,v_1,u_2,v_2} \mathbf{1}(u_1v_1m_{rs}l_{rr} > u_2v_2m_{rr}l_{rs})$$
$$\frac{p((i_1,j_1) \in E_{T+1}, (i_2,j_2) \notin E_{T+1}|(i_1,j_1) \in E_T, (i_2,j_2) \notin E_T, d_{i1,2} = u_{1,2}, d_{j1,2} = v_{1,2})}{p((i_1,j_1) \in E_T, (i_2,j_2) \notin E_T, (i_1,j_1) \in E_{T+1}, (i_2,j_2) \notin E_{T+1})}$$
$$\times p(d_{i1} = u_1)p(d_{j1} = v_1)p(d_{i2} = u_2)p(d_{j2} = v_2)$$

- Sixteenth term

$$
\begin{aligned}
&Pr(tes > tnes | te_{outside}, tne_{inside}, te_{non-edges}\, tne_{edges}) \\
&= \sum_{u_1,v_1,u_2,v_2} \mathbf{1}(u_1 v_1 m_{rs} l_{rs} > u_2 v_2 m_{rr} l_{rr}) \\
&\frac{p((i_1,j_1) \in E_{T+1}, (i_2,j_2) \notin E_{T+1} | (i_1,j_1) \notin E_T, (i_2,j_2) \in E_T, d_{i1,2} = u_{1,2}, d_{j1,2} = v_{1,2})}{p((i_1,j_1) \notin E_T, (i_2,j_2) \in E_T, (i_1,j_1) \in E_{T+1}, (i_2,j_2) \notin E_{T+1})} \\
&\times p(d_{i1} = u_1) p(d_{j1} = v_1) p(d_{i2} = u_2) p(d_{j2} = v_2)
\end{aligned}
$$

D: AUC, Precision, Recall

We here list the means and standard deviations of the AUC, Precision, and Recall scores(for balanced classes) for each of the different families of our link prediction experiments, comparing (and where possible combining) our Sequential-Stacking method with a tensorial method based on stochastic block modeling of multilayer networks (23) (which we label "Tensorial-SBM") and a network embedding deep learning framework, E-LSTM-D (24). We distinguish the cases where the target layer for the link prediction is partially observed from those where the target layer is completely unobserved, noting that the Tensorial-SBM method does not apply to the completely unobserved situation. The real-world datasets are the 19 considered elsewhere in the paper. Similarly, the community-label T-SBM and the edge-correlated T-SBM each consist of 45 realizations at different parameter values, as considered elsewhere in the paper.

Results for Real-World Data, Partially-Observed Target Layers			
Algorithm	AUC	Precision	Recall
Sequential-Stacking	0.917 ± 0.084	0.707 ± 0.167	0.794 ± 0.116
Sequential-Stacking with Tensorial SBM	0.921 ± 0.075	0.711 ± 0.164	0.798 ± 0.126
Sequential-Stacking with E-LSTM-D	0.911 ± 0.075	0.705 ± 0.16	0.801 ± 0.119
Sequential-Stacking with both	0.923 ± 0.083	0.701 ± 0.16	0.791 ± 0.128
Tensorial SBM	0.915 ± 0.086	0.697 ± 0.163	0.791 ± 0.129
E-LSTM-D (24)	0.859 ± 0.135	0.467 ± 0.163	0.605 ± 0.079

Results for Real-World Data, Completely-Unobserved Target Layers			
Algorithm	AUC	Precision	Recall
Sequential-Stacking	0.894 ± 0.131	0.823 ± 0.161	0.842 ± 0.148
Sequential-Stacking with E-LSTM-D	0.895 ± 0.129	0.821 ± 0.161	0.841 ± 0.151
E-LSTM-D	0.759 ± 0.135	0.816 ± 0.101	0.738 ± 0.132

Results for Community-Label T-SBM, Partially-Observed Target Layers			
Sequential-Stacking	0.818 ± 0.137	0.617 ± 0.173	0.735 ± 0.167
Sequential-Stacking with Tensorial SBM	0.818 ± 0.138	0.623 ± 0.171	0.733 ± 0.168
Sequential-Stacking with E-LSTM-D	0.818 ± 0.138	0.622 ± 0.173	0.736 ± 0.168
Sequential-Stacking with both	0.816 ± 0.14	0.623 ± 0.172	0.73 ± 0.167
Tensorial SBM	0.819 ± 0.138	0.621 ± 0.172	0.73 ± 0.17
E-LSTM-D	0.786 ± 0.014	0.673 ± 0.173	0.785 ± 0.14

Results for Community-Label T-SBM, Completely-Unobserved Target Layers			
Algorithm	AUC	Precision	Recall
Sequential-Stacking	0.758 ± 0.19	0.71 ± 0.211	0.739 ± 0.2
Sequential-Stacking with E-LSTM-D	0.759 ± 0.19	0.711 ± 0.211	0.74 ± 0.199
E-LSTM-D	0.606 ± 0.014	0.566 ± 0.06	0.67 ± 0.12

Results for Edge-Correlated T-SBM, Partially-Observed Target Layers			
Algorithm	AUC	Precision	Recall
Sequential-Stacking	0.915 ± 0.083	0.714 ± 0.15	0.837 ± 0.117
Sequential-Stacking with Tensorial SBM	0.914 ± 0.087	0.712 ± 0.151	0.84 ± 0.12
Sequential-Stacking with E-LSTM-D	0.917 ± 0.083	0.716 ± 0.149	0.844 ± 0.115
Sequential-Stacking with both	0.916 ± 0.083	0.717 ± 0.15	0.84 ± 0.12
Tensorial SBM	0.914 ± 0.087	0.715 ± 0.152	0.841 ± 0.122
E-LSTM-D	0.906 ± 0.062	0.773 ± 0.133	0.865 ± 0.094

Results for Edge-Correlated T-SBM, Completely-Unobserved Target Layers			
Algorithm	AUC	Precision	Recall
Sequential-Stacking	0.966 ± 0.032	0.914 ± 0.073	0.933 ± 0.057
Sequential-Stacking with E-LSTM-D	0.966 ± 0.033	0.912 ± 0.072	0.933 ± 0.056
E-LSTM-D	0.859 ± 0.062	0.867 ± 0.047	0.856 ± 0.037

AUC for Community-Label T-SBM, Partially-observed Target Layers					
Dataset	Top-Sequential-Stacking	TimeSeries	Tensorial-SBM	E-LSTM-D	Ensemble-Sequential-Stacking
p = 0.95, μ = 0.1, k = 1	0.695 ± 0.006	0.681 ± 0.006	0.636 ± 0.007	0.541 ± 0.007	0.705 ± 0.005
p = 0.95, μ = 0.1, k = 2	0.699 ± 0.005	0.713 ± 0.005	0.636 ± 0.003	0.538 ± 0.007	0.733 ± 0.004
p = 0.95, μ = 0.1, k = 5	0.836 ± 0.006	0.818 ± 0.007	0.635 ± 0.009	0.504 ± 0.006	0.84 ± 0.004
p = 0.95, μ = 0.1, k = 10	0.905 ± 0.005	0.902 ± 0.004	0.558 ± 0.005	0.605 ± 0.009	0.918 ± 0.004
p = 0.95, μ = 0.2, k = 1	0.681 ± 0.005	0.661 ± 0.004	0.574 ± 0.002	0.54 ± 0.005	0.664 ± 0.006
p = 0.95, μ = 0.2, k = 2	0.653 ± 0.007	0.668 ± 0.003	0.565 ± 0.003	0.544 ± 0.008	0.689 ± 0.005
p = 0.95, μ = 0.2, k = 5	0.793 ± 0.003	0.781 ± 0.002	0.559 ± 0.011	0.581 ± 0.01	0.813 ± 0.005
p = 0.95, μ = 0.2, k = 10	0.851 ± 0.005	0.835 ± 0.005	0.604 ± 0.006	0.521 ± 0.007	0.866 ± 0.006
p = 0.95, μ = 0.3, k = 1	0.703 ± 0.005	0.679 ± 0.006	0.564 ± 0.005	0.516 ± 0.012	0.698 ± 0.009
p = 0.95, μ = 0.3, k = 2	0.685 ± 0.005	0.672 ± 0.006	0.623 ± 0.003	0.508 ± 0.01	0.718 ± 0.006
p = 0.95, μ = 0.3, k = 5	0.744 ± 0.007	0.731 ± 0.004	0.584 ± 0.005	0.521 ± 0.011	0.751 ± 0.008
p = 0.95, μ = 0.3, k = 10	0.766 ± 0.005	0.766 ± 0.006	0.588 ± 0.003	0.489 ± 0.006	0.781 ± 0.007
p = 0.85, μ = 0.1, k = 1	0.678 ± 0.004	0.666 ± 0.003	0.636 ± 0.006	0.568 ± 0.012	0.697 ± 0.006
p = 0.85, μ = 0.1, k = 2	0.729 ± 0.004	0.705 ± 0.006	0.597 ± 0.006	0.524 ± 0.007	0.744 ± 0.005
p = 0.85, μ = 0.1, k = 5	0.878 ± 0.003	0.861 ± 0.006	0.523 ± 0.006	0.491 ± 0.008	0.883 ± 0.004
p = 0.85, μ = 0.1, k = 10	0.887 ± 0.005	0.879 ± 0.005	0.517 ± 0.005	0.549 ± 0.008	0.891 ± 0.006
p = 0.85, μ = 0.2, k = 1	0.677 ± 0.008	0.654 ± 0.003	0.574 ± 0.005	0.547 ± 0.005	0.672 ± 0.004
p = 0.85, μ = 0.2, k = 2	0.677 ± 0.007	0.67 ± 0.005	0.59 ± 0.005	0.543 ± 0.006	0.672 ± 0.005
p = 0.85, μ = 0.2, k = 5	0.811 ± 0.005	0.815 ± 0.005	0.534 ± 0.002	0.53 ± 0.006	0.834 ± 0.006
p = 0.85, μ = 0.2, k = 10	0.845 ± 0.005	0.814 ± 0.004	0.597 ± 0.004	0.521 ± 0.009	0.864 ± 0.005
p = 0.85, μ = 0.3, k = 1	0.67 ± 0.005	0.651 ± 0.005	0.619 ± 0.003	0.503 ± 0.008	0.683 ± 0.005
p = 0.85, μ = 0.3, k = 2	0.682 ± 0.005	0.68 ± 0.007	0.51 ± 0.005	0.507 ± 0.011	0.688 ± 0.005
p = 0.85, μ = 0.3, k = 5	0.738 ± 0.005	0.714 ± 0.006	0.53 ± 0.003	0.542 ± 0.009	0.739 ± 0.004
p = 0.85, μ = 0.3, k = 10	0.832 ± 0.004	0.828 ± 0.004	0.592 ± 0.005	0.563 ± 0.01	0.847 ± 0.006
p = 0.75, μ = 0.1, k = 1	0.648 ± 0.006	0.64 ± 0.003	0.58 ± 0.007	0.497 ± 0.006	0.666 ± 0.004
p = 0.75, μ = 0.1, k = 2	0.71 ± 0.005	0.727 ± 0.006	0.535 ± 0.004	0.519 ± 0.01	0.739 ± 0.005
p = 0.75, μ = 0.1, k = 5	0.833 ± 0.004	0.802 ± 0.006	0.589 ± 0.008	0.486 ± 0.01	0.841 ± 0.004
p = 0.75, μ = 0.1, k = 10	0.917 ± 0.003	0.914 ± 0.005	0.555 ± 0.005	0.527 ± 0.005	0.926 ± 0.008
p = 0.75, μ = 0.2, k = 1	0.724 ± 0.008	0.696 ± 0.006	0.605 ± 0.004	0.559 ± 0.008	0.708 ± 0.008
p = 0.75, μ = 0.2, k = 2	0.651 ± 0.003	0.637 ± 0.007	0.559 ± 0.006	0.512 ± 0.006	0.66 ± 0.005
p = 0.75, μ = 0.2, k = 5	0.803 ± 0.005	0.803 ± 0.004	0.575 ± 0.005	0.545 ± 0.017	0.821 ± 0.006
p = 0.75, μ = 0.2, k = 10	0.838 ± 0.006	0.83 ± 0.007	0.58 ± 0.009	0.49 ± 0.008	0.854 ± 0.004
p = 0.75, μ = 0.3, k = 1	0.703 ± 0.006	0.682 ± 0.006	0.564 ± 0.007	0.504 ± 0.006	0.703 ± 0.004
p = 0.75, μ = 0.3, k = 2	0.692 ± 0.006	0.674 ± 0.004	0.614 ± 0.005	0.516 ± 0.01	0.707 ± 0.006
p = 0.75, μ = 0.3, k = 5	0.769 ± 0.004	0.773 ± 0.006	0.55 ± 0.004	0.526 ± 0.011	0.784 ± 0.004
p = 0.75, μ = 0.3, k = 10	0.78 ± 0.004	0.775 ± 0.005	0.564 ± 0.005	0.559 ± 0.008	0.789 ± 0.004
p = 0.95, μ = 0.1, k = 15	0.907 ± 0.006	0.909 ± 0.004	0.549 ± 0.003	0.513 ± 0.008	0.915 ± 0.004
p = 0.95, μ = 0.2, k = 15	0.859 ± 0.007	0.85 ± 0.006	0.531 ± 0.004	0.459 ± 0.008	0.871 ± 0.006
p = 0.95, μ = 0.3, k = 15	0.82 ± 0.004	0.779 ± 0.009	0.544 ± 0.004	0.551 ± 0.008	0.833 ± 0.005
p = 0.85, μ = 0.1, k = 15	0.887 ± 0.004	0.872 ± 0.004	0.547 ± 0.007	0.578 ± 0.01	0.908 ± 0.004
p = 0.85, μ = 0.2, k = 15	0.847 ± 0.004	0.824 ± 0.004	0.499 ± 0.006	0.5 ± 0.01	0.863 ± 0.004
p = 0.85, μ = 0.3, k = 15	0.795 ± 0.003	0.765 ± 0.005	0.556 ± 0.005	0.494 ± 0.006	0.824 ± 0.008
p = 0.75, μ = 0.1, k = 15	0.907 ± 0.004	0.897 ± 0.004	0.562 ± 0.005	0.442 ± 0.006	0.916 ± 0.005
p = 0.75, μ = 0.2, k = 15	0.819 ± 0.005	0.79 ± 0.005	0.489 ± 0.007	0.563 ± 0.006	0.837 ± 0.006
p = 0.75, μ = 0.3, k = 15	0.814 ± 0.005	0.754 ± 0.002	0.493 ± 0.006	0.552 ± 0.012	0.814 ± 0.004

AUC for Community-Label T-SBM, Completely-unobserved Target Layers				
Dataset	Top-Sequential-Stacking	TimeSeries	E-LSTM-D	Ensemble-Sequential-Stacking
p = 0.95, μ = 0.1, k = 1	0.543 ± 0.01	0.457 ± 0.015	0.521 ± 0.01	0.623 ± 0.009
p = 0.95, μ = 0.1, k = 2	0.677 ± 0.011	0.628 ± 0.01	0.5 ± 0.013	0.687 ± 0.01
p = 0.95, μ = 0.1, k = 5	0.734 ± 0.004	0.71 ± 0.008	0.526 ± 0.012	0.757 ± 0.011
p = 0.95, μ = 0.1, k = 10	0.819 ± 0.005	0.774 ± 0.005	0.519 ± 0.007	0.82 ± 0.006
p = 0.95, μ = 0.2, k = 1	0.48 ± 0.011	0.473 ± 0.005	0.569 ± 0.008	0.551 ± 0.007
p = 0.95, μ = 0.2, k = 2	0.593 ± 0.007	0.578 ± 0.014	0.458 ± 0.011	0.608 ± 0.011
p = 0.95, μ = 0.2, k = 5	0.693 ± 0.004	0.666 ± 0.005	0.561 ± 0.01	0.705 ± 0.009
p = 0.95, μ = 0.2, k = 10	0.787 ± 0.006	0.777 ± 0.007	0.546 ± 0.006	0.8 ± 0.006
p = 0.95, μ = 0.3, k = 1	0.543 ± 0.01	0.51 ± 0.009	0.509 ± 0.012	0.549 ± 0.008
p = 0.95, μ = 0.3, k = 2	0.585 ± 0.008	0.56 ± 0.009	0.516 ± 0.013	0.642 ± 0.01
p = 0.95, μ = 0.3, k = 5	0.635 ± 0.008	0.616 ± 0.005	0.479 ± 0.007	0.653 ± 0.007
p = 0.95, μ = 0.3, k = 10	0.717 ± 0.004	0.695 ± 0.007	0.506 ± 0.009	0.733 ± 0.009
p = 0.85, μ = 0.1, k = 1	0.484 ± 0.009	0.5 ± 0.011	0.529 ± 0.009	0.584 ± 0.012
p = 0.85, μ = 0.1, k = 2	0.635 ± 0.005	0.564 ± 0.014	0.555 ± 0.007	0.634 ± 0.011
p = 0.85, μ = 0.1, k = 5	0.762 ± 0.009	0.711 ± 0.012	0.503 ± 0.013	0.754 ± 0.011
p = 0.85, μ = 0.1, k = 10	0.757 ± 0.007	0.731 ± 0.006	0.517 ± 0.012	0.751 ± 0.005
p = 0.85, μ = 0.2, k = 1	0.509 ± 0.014	0.521 ± 0.013	0.532 ± 0.007	0.56 ± 0.011
p = 0.85, μ = 0.2, k = 2	0.563 ± 0.008	0.499 ± 0.015	0.527 ± 0.009	0.565 ± 0.008
p = 0.85, μ = 0.2, k = 5	0.692 ± 0.008	0.635 ± 0.008	0.493 ± 0.005	0.687 ± 0.008
p = 0.85, μ = 0.2, k = 10	0.725 ± 0.011	0.708 ± 0.007	0.556 ± 0.006	0.744 ± 0.006
p = 0.85, μ = 0.3, k = 1	0.506 ± 0.012	0.478 ± 0.012	0.514 ± 0.007	0.565 ± 0.007
p = 0.85, μ = 0.3, k = 2	0.556 ± 0.006	0.505 ± 0.009	0.458 ± 0.017	0.561 ± 0.009
p = 0.85, μ = 0.3, k = 5	0.583 ± 0.006	0.53 ± 0.017	0.566 ± 0.01	0.589 ± 0.009
p = 0.85, μ = 0.3, k = 10	0.774 ± 0.004	0.724 ± 0.008	0.508 ± 0.012	0.771 ± 0.008
p = 0.75, μ = 0.1, k = 1	0.52 ± 0.01	0.521 ± 0.012	0.532 ± 0.007	0.612 ± 0.009
p = 0.75, μ = 0.1, k = 2	0.569 ± 0.006	0.548 ± 0.009	0.455 ± 0.011	0.608 ± 0.01
p = 0.75, μ = 0.1, k = 5	0.669 ± 0.014	0.616 ± 0.006	0.503 ± 0.01	0.683 ± 0.01
p = 0.75, μ = 0.1, k = 10	0.701 ± 0.004	0.689 ± 0.015	0.525 ± 0.009	0.722 ± 0.009
p = 0.75, μ = 0.2, k = 1	0.526 ± 0.009	0.525 ± 0.012	0.498 ± 0.01	0.562 ± 0.008
p = 0.75, μ = 0.2, k = 2	0.508 ± 0.012	0.496 ± 0.009	0.503 ± 0.014	0.517 ± 0.008
p = 0.75, μ = 0.2, k = 5	0.624 ± 0.008	0.539 ± 0.009	0.486 ± 0.009	0.634 ± 0.009
p = 0.75, μ = 0.2, k = 10	0.645 ± 0.007	0.64 ± 0.008	0.487 ± 0.011	0.678 ± 0.009
p = 0.75, μ = 0.3, k = 1	0.492 ± 0.011	0.524 ± 0.007	0.505 ± 0.014	0.558 ± 0.005
p = 0.75, μ = 0.3, k = 2	0.535 ± 0.008	0.549 ± 0.012	0.518 ± 0.008	0.588 ± 0.008
p = 0.75, μ = 0.3, k = 5	0.598 ± 0.007	0.577 ± 0.011	0.535 ± 0.011	0.589 ± 0.008
p = 0.75, μ = 0.3, k = 10	0.646 ± 0.005	0.603 ± 0.007	0.508 ± 0.009	0.663 ± 0.008
p = 0.95, μ = 0.1, k = 15	0.842 ± 0.003	0.827 ± 0.005	0.487 ± 0.005	0.822 ± 0.005
p = 0.95, μ = 0.2, k = 15	0.833 ± 0.005	0.835 ± 0.006	0.577 ± 0.008	0.836 ± 0.005
p = 0.95, μ = 0.3, k = 15	0.754 ± 0.007	0.726 ± 0.01	0.507 ± 0.007	0.76 ± 0.003
p = 0.85, μ = 0.1, k = 15	0.745 ± 0.006	0.768 ± 0.006	0.514 ± 0.009	0.778 ± 0.006
p = 0.85, μ = 0.2, k = 15	0.75 ± 0.006	0.701 ± 0.005	0.509 ± 0.01	0.727 ± 0.005
p = 0.85, μ = 0.3, k = 15	0.673 ± 0.006	0.68 ± 0.007	0.502 ± 0.009	0.703 ± 0.008
p = 0.75, μ = 0.1, k = 15	0.625 ± 0.005	0.647 ± 0.004	0.522 ± 0.01	0.72 ± 0.006
p = 0.75, μ = 0.2, k = 15	0.667 ± 0.011	0.703 ± 0.01	0.545 ± 0.011	0.717 ± 0.008
p = 0.75, μ = 0.3, k = 15	0.687 ± 0.009	0.663 ± 0.008	0.502 ± 0.005	0.679 ± 0.01

AUC for Edge-Correlated T-SBM, Partially-observed Target Layers					
Dataset	Top-Sequential-Stacking	TimeSeries	Tensorial-SBM	E-LSTM-D	Ensemble-Sequential-Stacking
p = 0.8, μ = 0.1, k = 1	0.936 ± 0.004	0.891 ± 0.007	0.642 ± 0.002	0.579 ± 0.009	0.94 ± 0.002
p = 0.8, μ = 0.1, k = 2	0.951 ± 0.003	0.902 ± 0.01	0.708 ± 0.004	0.593 ± 0.008	0.952 ± 0.003
p = 0.8, μ = 0.1, k = 5	0.979 ± 0.004	0.922 ± 0.005	0.827 ± 0.004	0.631 ± 0.005	0.98 ± 0.004
p = 0.8, μ = 0.1, k = 10	0.982 ± 0.004	0.949 ± 0.004	0.836 ± 0.002	0.609 ± 0.005	0.986 ± 0.004
p = 0.8, μ = 0.2, k = 1	0.936 ± 0.005	0.915 ± 0.01	0.66 ± 0.004	0.593 ± 0.003	0.937 ± 0.003
p = 0.8, μ = 0.2, k = 2	0.969 ± 0.003	0.9 ± 0.008	0.781 ± 0.005	0.605 ± 0.005	0.97 ± 0.004
p = 0.8, μ = 0.2, k = 5	0.978 ± 0.004	0.932 ± 0.006	0.81 ± 0.004	0.568 ± 0.008	0.978 ± 0.003
p = 0.8, μ = 0.2, k = 10	0.976 ± 0.002	0.933 ± 0.006	0.814 ± 0.005	0.651 ± 0.006	0.976 ± 0.003
p = 0.8, μ = 0.3, k = 1	0.939 ± 0.003	0.901 ± 0.011	0.663 ± 0.008	0.625 ± 0.004	0.943 ± 0.003
p = 0.8, μ = 0.3, k = 2	0.958 ± 0.003	0.924 ± 0.009	0.647 ± 0.003	0.595 ± 0.004	0.959 ± 0.003
p = 0.8, μ = 0.3, k = 5	0.954 ± 0.003	0.901 ± 0.008	0.751 ± 0.004	0.573 ± 0.009	0.958 ± 0.004
p = 0.8, μ = 0.3, k = 10	0.962 ± 0.004	0.921 ± 0.007	0.812 ± 0.006	0.659 ± 0.006	0.968 ± 0.003
p = 0.7, μ = 0.1, k = 1	0.917 ± 0.003	0.867 ± 0.008	0.669 ± 0.005	0.593 ± 0.003	0.921 ± 0.004
p = 0.7, μ = 0.1, k = 2	0.947 ± 0.005	0.889 ± 0.007	0.765 ± 0.005	0.534 ± 0.004	0.952 ± 0.004
p = 0.7, μ = 0.1, k = 5	0.968 ± 0.003	0.945 ± 0.004	0.809 ± 0.005	0.618 ± 0.008	0.973 ± 0.004
p = 0.7, μ = 0.1, k = 10	0.974 ± 0.003	0.921 ± 0.005	0.708 ± 0.003	0.584 ± 0.005	0.978 ± 0.003
p = 0.7, μ = 0.2, k = 1	0.909 ± 0.004	0.818 ± 0.009	0.688 ± 0.004	0.649 ± 0.004	0.908 ± 0.004
p = 0.7, μ = 0.2, k = 2	0.938 ± 0.004	0.882 ± 0.01	0.794 ± 0.006	0.687 ± 0.006	0.944 ± 0.005
p = 0.7, μ = 0.2, k = 5	0.934 ± 0.004	0.859 ± 0.007	0.66 ± 0.004	0.632 ± 0.005	0.938 ± 0.004
p = 0.7, μ = 0.2, k = 10	0.964 ± 0.003	0.909 ± 0.006	0.814 ± 0.003	0.636 ± 0.007	0.968 ± 0.004
p = 0.7, μ = 0.3, k = 1	0.894 ± 0.004	0.847 ± 0.008	0.672 ± 0.005	0.572 ± 0.004	0.898 ± 0.005
p = 0.7, μ = 0.3, k = 2	0.928 ± 0.004	0.839 ± 0.007	0.708 ± 0.006	0.602 ± 0.008	0.932 ± 0.004
p = 0.7, μ = 0.3, k = 5	0.924 ± 0.003	0.878 ± 0.009	0.727 ± 0.004	0.642 ± 0.006	0.93 ± 0.004
p = 0.7, μ = 0.3, k = 10	0.953 ± 0.003	0.894 ± 0.007	0.814 ± 0.002	0.552 ± 0.011	0.957 ± 0.004
p = 0.6, μ = 0.1, k = 1	0.884 ± 0.005	0.824 ± 0.007	0.734 ± 0.004	0.603 ± 0.007	0.893 ± 0.004
p = 0.6, μ = 0.1, k = 2	0.918 ± 0.005	0.847 ± 0.008	0.776 ± 0.003	0.613 ± 0.007	0.925 ± 0.006
p = 0.6, μ = 0.1, k = 5	0.944 ± 0.005	0.897 ± 0.006	0.86 ± 0.005	0.653 ± 0.003	0.955 ± 0.006
p = 0.6, μ = 0.1, k = 10	0.966 ± 0.003	0.937 ± 0.005	0.79 ± 0.003	0.595 ± 0.006	0.968 ± 0.004
p = 0.6, μ = 0.2, k = 1	0.864 ± 0.004	0.818 ± 0.005	0.656 ± 0.004	0.652 ± 0.006	0.871 ± 0.004
p = 0.6, μ = 0.2, k = 2	0.898 ± 0.004	0.835 ± 0.007	0.756 ± 0.006	0.584 ± 0.008	0.908 ± 0.004
p = 0.6, μ = 0.2, k = 5	0.942 ± 0.003	0.9 ± 0.006	0.793 ± 0.002	0.588 ± 0.005	0.948 ± 0.006
p = 0.6, μ = 0.2, k = 10	0.937 ± 0.003	0.886 ± 0.006	0.793 ± 0.002	0.62 ± 0.007	0.946 ± 0.004
p = 0.6, μ = 0.3, k = 1	0.86 ± 0.005	0.792 ± 0.006	0.689 ± 0.005	0.599 ± 0.008	0.864 ± 0.005
p = 0.6, μ = 0.3, k = 2	0.902 ± 0.005	0.815 ± 0.006	0.717 ± 0.007	0.556 ± 0.006	0.903 ± 0.004
p = 0.6, μ = 0.3, k = 5	0.915 ± 0.005	0.853 ± 0.008	0.687 ± 0.008	0.6 ± 0.006	0.923 ± 0.003
p = 0.6, μ = 0.3, k = 10	0.909 ± 0.006	0.836 ± 0.007	0.715 ± 0.002	0.598 ± 0.004	0.917 ± 0.004
p = 0.8, μ = 0.1, k = 15	0.989 ± 0.003	0.951 ± 0.005	0.832 ± 0.004	0.664 ± 0.008	0.99 ± 0.004
p = 0.8, μ = 0.2, k = 15	0.986 ± 0.003	0.941 ± 0.007	0.748 ± 0.003	0.666 ± 0.005	0.987 ± 0.006
p = 0.8, μ = 0.3, k = 15	0.978 ± 0.002	0.934 ± 0.008	0.736 ± 0.003	0.625 ± 0.005	0.983 ± 0.005
p = 0.7, μ = 0.1, k = 15	0.979 ± 0.004	0.958 ± 0.004	0.706 ± 0.007	0.687 ± 0.003	0.982 ± 0.004
p = 0.7, μ = 0.2, k = 15	0.953 ± 0.004	0.916 ± 0.005	0.705 ± 0.003	0.606 ± 0.006	0.957 ± 0.004
p = 0.7, μ = 0.3, k = 15	0.961 ± 0.002	0.89 ± 0.008	0.756 ± 0.006	0.587 ± 0.006	0.963 ± 0.003
p = 0.6, μ = 0.1, k = 15	0.959 ± 0.004	0.938 ± 0.006	0.852 ± 0.005	0.581 ± 0.008	0.964 ± 0.003
p = 0.6, μ = 0.2, k = 15	0.96 ± 0.003	0.927 ± 0.005	0.778 ± 0.004	0.553 ± 0.007	0.967 ± 0.004
p = 0.6, μ = 0.3, k = 15	0.921 ± 0.004	0.84 ± 0.008	0.738 ± 0.004	0.584 ± 0.005	0.93 ± 0.003

AUC for Edge-Correlated T-SBM, Completely-unobserved Target Layers				
Dataset	Top-Sequential-Stacking	TimeSeries	E-LSTM-D	Ensemble-Sequential-Stacking
p = 0.8, μ = 0.1, k = 1	0.942 ± 0.003	0.937 ± 0.002	0.571 ± 0.01	0.942 ± 0.003
p = 0.8, μ = 0.1, k = 2	0.957 ± 0.001	0.951 ± 0.003	0.605 ± 0.006	0.956 ± 0.002
p = 0.8, μ = 0.1, k = 5	0.98 ± 0.001	0.977 ± 0.002	0.641 ± 0.01	0.981 ± 0.001
p = 0.8, μ = 0.1, k = 10	0.986 ± 0.002	0.982 ± 0.002	0.606 ± 0.013	0.987 ± 0.001
p = 0.8, μ = 0.2, k = 1	0.94 ± 0.003	0.935 ± 0.005	0.586 ± 0.01	0.939 ± 0.002
p = 0.8, μ = 0.2, k = 2	0.971 ± 0.003	0.966 ± 0.002	0.577 ± 0.006	0.971 ± 0.002
p = 0.8, μ = 0.2, k = 5	0.977 ± 0.001	0.975 ± 0.002	0.586 ± 0.008	0.978 ± 0.002
p = 0.8, μ = 0.2, k = 10	0.976 ± 0.001	0.972 ± 0.002	0.586 ± 0.007	0.973 ± 0.002
p = 0.8, μ = 0.3, k = 1	0.943 ± 0.002	0.939 ± 0.002	0.574 ± 0.01	0.943 ± 0.002
p = 0.8, μ = 0.3, k = 2	0.961 ± 0.002	0.956 ± 0.003	0.582 ± 0.008	0.959 ± 0.003
p = 0.8, μ = 0.3, k = 5	0.959 ± 0.003	0.951 ± 0.002	0.546 ± 0.009	0.96 ± 0.002
p = 0.8, μ = 0.3, k = 10	0.968 ± 0.003	0.968 ± 0.003	0.581 ± 0.011	0.968 ± 0.002
p = 0.7, μ = 0.1, k = 1	0.925 ± 0.002	0.916 ± 0.007	0.634 ± 0.007	0.925 ± 0.004
p = 0.7, μ = 0.1, k = 2	0.95 ± 0.003	0.938 ± 0.002	0.594 ± 0.008	0.949 ± 0.004
p = 0.7, μ = 0.1, k = 5	0.971 ± 0.004	0.965 ± 0.002	0.593 ± 0.007	0.973 ± 0.002
p = 0.7, μ = 0.1, k = 10	0.979 ± 0.002	0.976 ± 0.002	0.659 ± 0.005	0.979 ± 0.001
p = 0.7, μ = 0.2, k = 1	0.918 ± 0.003	0.906 ± 0.005	0.588 ± 0.013	0.917 ± 0.004
p = 0.7, μ = 0.2, k = 2	0.944 ± 0.004	0.93 ± 0.003	0.567 ± 0.008	0.944 ± 0.002
p = 0.7, μ = 0.2, k = 5	0.941 ± 0.002	0.928 ± 0.005	0.597 ± 0.006	0.938 ± 0.002
p = 0.7, μ = 0.2, k = 10	0.965 ± 0.004	0.956 ± 0.003	0.662 ± 0.005	0.963 ± 0.002
p = 0.7, μ = 0.3, k = 1	0.899 ± 0.002	0.889 ± 0.002	0.572 ± 0.009	0.898 ± 0.003
p = 0.7, μ = 0.3, k = 2	0.934 ± 0.004	0.925 ± 0.003	0.613 ± 0.008	0.935 ± 0.005
p = 0.7, μ = 0.3, k = 5	0.932 ± 0.003	0.919 ± 0.002	0.584 ± 0.008	0.932 ± 0.003
p = 0.7, μ = 0.3, k = 10	0.959 ± 0.003	0.952 ± 0.003	0.611 ± 0.012	0.958 ± 0.003
p = 0.6, μ = 0.1, k = 1	0.895 ± 0.003	0.882 ± 0.004	0.649 ± 0.006	0.895 ± 0.008
p = 0.6, μ = 0.1, k = 2	0.927 ± 0.002	0.912 ± 0.004	0.611 ± 0.01	0.925 ± 0.004
p = 0.6, μ = 0.1, k = 5	0.955 ± 0.002	0.945 ± 0.003	0.574 ± 0.008	0.956 ± 0.002
p = 0.6, μ = 0.1, k = 10	0.968 ± 0.003	0.963 ± 0.001	0.633 ± 0.005	0.969 ± 0.001
p = 0.6, μ = 0.2, k = 1	0.875 ± 0.007	0.858 ± 0.003	0.573 ± 0.011	0.873 ± 0.004
p = 0.6, μ = 0.2, k = 2	0.91 ± 0.004	0.891 ± 0.003	0.696 ± 0.007	0.909 ± 0.003
p = 0.6, μ = 0.2, k = 5	0.951 ± 0.003	0.942 ± 0.002	0.596 ± 0.01	0.949 ± 0.002
p = 0.6, μ = 0.2, k = 10	0.95 ± 0.003	0.945 ± 0.003	0.685 ± 0.007	0.951 ± 0.003
p = 0.6, μ = 0.3, k = 1	0.868 ± 0.004	0.856 ± 0.003	0.59 ± 0.008	0.868 ± 0.004
p = 0.6, μ = 0.3, k = 2	0.909 ± 0.004	0.892 ± 0.005	0.583 ± 0.009	0.909 ± 0.003
p = 0.6, μ = 0.3, k = 5	0.923 ± 0.005	0.907 ± 0.008	0.61 ± 0.006	0.922 ± 0.004
p = 0.6, μ = 0.3, k = 10	0.92 ± 0.002	0.904 ± 0.004	0.604 ± 0.007	0.918 ± 0.002
p = 0.8, μ = 0.1, k = 15	0.99 ± 0.002	0.987 ± 0.002	0.589 ± 0.006	0.989 ± 0.001
p = 0.8, μ = 0.2, k = 15	0.99 ± 0.003	0.987 ± 0.002	0.58 ± 0.013	0.989 ± 0.002
p = 0.8, μ = 0.3, k = 15	0.983 ± 0.003	0.976 ± 0.003	0.592 ± 0.01	0.982 ± 0.002
p = 0.7, μ = 0.1, k = 15	0.986 ± 0.002	0.982 ± 0.002	0.662 ± 0.008	0.985 ± 0.002
p = 0.7, μ = 0.2, k = 15	0.959 ± 0.002	0.951 ± 0.003	0.614 ± 0.009	0.956 ± 0.002
p = 0.7, μ = 0.3, k = 15	0.968 ± 0.005	0.959 ± 0.004	0.598 ± 0.014	0.968 ± 0.002
p = 0.6, μ = 0.1, k = 15	0.968 ± 0.002	0.962 ± 0.002	0.729 ± 0.003	0.967 ± 0.002
p = 0.6, μ = 0.2, k = 15	0.963 ± 0.003	0.959 ± 0.003	0.618 ± 0.006	0.964 ± 0.001
p = 0.6, μ = 0.3, k = 15	0.929 ± 0.004	0.918 ± 0.003	0.579 ± 0.004	0.931 ± 0.003

AUC for Real-World Networks, Partially-observed Target Layers					
Dataset	Top-Sequential-Stacking	TimeSeries	Tensorial-SBM	E-LSTM-D	Ensemble-Sequential-Stacking
chess	0.995 ± 0.002	0.998 ± 0.003	0.75 ± 0.002	0.747 ± 0.003	0.996 ± 0.003
obrazil	0.83 ± 0.005	0.733 ± 0.003	0.466 ± 0.003	0.555 ± 0.005	0.862 ± 0.006
bionet1	0.903 ± 0.004	0.917 ± 0.003	0.848 ± 0.002	0.847 ± 0.007	0.915 ± 0.003
bitcoin	0.992 ± 0.004	0.993 ± 0.003	0.963 ± 0.002	0.991 ± 0.001	0.994 ± 0.002
emaildnc	0.994 ± 0.003	0.987 ± 0.002	0.996 ± 0.002	0.989 ± 0.0	0.951 ± 0.0
bionet2	0.882 ± 0.005	0.91 ± 0.003	0.87 ± 0.003	0.881 ± 0.004	0.91 ± 0.004
obitcoin	0.801 ± 0.004	0.79 ± 0.002	0.528 ± 0.001	0.597 ± 0.003	0.831 ± 0.004
london	0.73 ± 0.006	0.769 ± 0.003	0.701 ± 0.004	0.68 ± 0.01	0.739 ± 0.003
collegemsg	0.924 ± 0.004	0.926 ± 0.004	0.845 ± 0.002	0.96 ± 0.002	0.958 ± 0.003
fbmsg	0.975 ± 0.003	0.949 ± 0.003	0.801 ± 0.002	0.98 ± 0.003	0.984 ± 0.003
radoslaw	0.97 ± 0.004	0.937 ± 0.003	0.878 ± 0.002	0.855 ± 0.004	0.972 ± 0.004
fbforum	0.961 ± 0.003	0.923 ± 0.004	0.929 ± 0.003	0.951 ± 0.003	0.979 ± 0.003
mit	0.96 ± 0.003	0.92 ± 0.004	0.871 ± 0.004	0.87 ± 0.004	0.984 ± 0.002
ant1	0.929 ± 0.005	0.831 ± 0.006	0.808 ± 0.007	0.596 ± 0.004	0.935 ± 0.003
ant2	0.869 ± 0.007	0.816 ± 0.003	0.802 ± 0.005	0.687 ± 0.006	0.883 ± 0.004
ant3	0.88 ± 0.005	0.797 ± 0.003	0.843 ± 0.007	0.675 ± 0.008	0.892 ± 0.004
ant4	0.928 ± 0.005	0.851 ± 0.005	0.865 ± 0.006	0.614 ± 0.005	0.936 ± 0.004
ant5	0.878 ± 0.007	0.828 ± 0.003	0.812 ± 0.007	0.735 ± 0.004	0.877 ± 0.004
ant6	0.885 ± 0.006	0.804 ± 0.005	0.808 ± 0.007	0.707 ± 0.008	0.893 ± 0.004

AUC for Real-World Networks, Completely-unobserved Target Layers				
Dataset	Top-Sequential-Stacking	TimeSeries	E-LSTM-D	Ensemble-Sequential-Stacking
chess	0.514 ± 0.002	0.601 ± 0.003	0.63 ± 0.003	0.649 ± 0.003
obrazil	0.832 ± 0.005	0.783 ± 0.003	0.56 ± 0.005	0.86 ± 0.006
bionet1	0.817 ± 0.004	0.852 ± 0.003	0.842 ± 0.007	0.849 ± 0.003
bitcoin	0.801 ± 0.004	0.967 ± 0.003	0.97 ± 0.001	0.947 ± 0.002
emaildnc	0.905 ± 0.003	0.946 ± 0.002	0.908 ± 0.0	0.996 ± 0.0
bionet2	0.847 ± 0.005	0.86 ± 0.003	0.875 ± 0.004	0.867 ± 0.004
obitcoin	0.634 ± 0.004	0.53 ± 0.002	0.515 ± 0.003	0.606 ± 0.004
london	0.63 ± 0.006	0.676 ± 0.003	0.678 ± 0.01	0.706 ± 0.003
collegemsg	0.921 ± 0.004	0.94 ± 0.004	0.929 ± 0.002	0.95 ± 0.003
fbmsg	0.945 ± 0.003	0.927 ± 0.003	0.913 ± 0.003	0.944 ± 0.003
radoslaw	0.974 ± 0.004	0.965 ± 0.003	0.887 ± 0.004	0.972 ± 0.004
fbforum	0.96 ± 0.003	0.955 ± 0.004	0.899 ± 0.003	0.967 ± 0.003
mit	0.976 ± 0.003	0.949 ± 0.004	0.876 ± 0.004	0.978 ± 0.002
ant1	0.929 ± 0.005	0.926 ± 0.006	0.801 ± 0.004	0.931 ± 0.003
ant2	0.85 ± 0.007	0.846 ± 0.003	0.8 ± 0.006	0.848 ± 0.004
ant3	0.88 ± 0.005	0.861 ± 0.003	0.805 ± 0.008	0.879 ± 0.004
ant4	0.926 ± 0.005	0.922 ± 0.005	0.839 ± 0.005	0.924 ± 0.004
ant5	0.863 ± 0.007	0.855 ± 0.003	0.809 ± 0.004	0.863 ± 0.004
ant6	0.876 ± 0.006	0.863 ± 0.005	0.785 ± 0.008	0.875 ± 0.004

E: Individual Dataset AUC, Auto-Correlation.

Results for Real-world Networks	
Dataset Name	Auto Correlation Score
chess	-82.367
obrazil	-64.204
bionet1	-209.469
bitcoin	-63.184
emaildnc	32.857
bionet2	-252.857
obitcoin	-64.98
london	-514.612
collegemsg	-294.98
fbmsg	-270.163
radoslaw	3445.878
fbforum	817.551
mit	1065.592
ant1	10790.49
ant2	26379.265
ant3	37531.51
ant4	9909.959
ant5	33823.184
ant6	43593.551

Results for Community-Label T-SBM Networks	
Dataset Name	Auto Correlation Score
p = 0.95, μ = 0.1, k = 1	-2.49
p = 0.95, μ = 0.1, k = 2	65.796
p = 0.95, μ = 0.1, k = 5	186.449
p = 0.95, μ = 0.1, k = 10	406.286
p = 0.95, μ = 0.2, k = 1	-37.224
p = 0.95, μ = 0.2, k = 2	1.469
p = 0.95, μ = 0.2, k = 5	148.408
p = 0.95, μ = 0.2, k = 10	506.531
p = 0.95, μ = 0.3, k = 1	-41.918
p = 0.95, μ = 0.3, k = 2	-17.143
p = 0.95, μ = 0.3, k = 5	171.551
p = 0.95, μ = 0.3, k = 10	307.551
p = 0.85, μ = 0.1, k = 1	-47.796
p = 0.85, μ = 0.1, k = 2	15.184
p = 0.85, μ = 0.1, k = 5	67.673
p = 0.85, μ = 0.1, k = 10	118.735
p = 0.85, μ = 0.2, k = 1	-24.816
p = 0.85, μ = 0.2, k = 2	-37.429
p = 0.85, μ = 0.2, k = 5	56.898
p = 0.85, μ = 0.2, k = 10	185.02
p = 0.85, μ = 0.3, k = 1	-40.367
p = 0.85, μ = 0.3, k = 2	-21.878
p = 0.85, μ = 0.3, k = 5	3.02
p = 0.85, μ = 0.3, k = 10	36.286
p = 0.75, μ = 0.1, k = 1	-33.918
p = 0.75, μ = 0.1, k = 2	-24.408
p = 0.75, μ = 0.1, k = 5	49.347
p = 0.75, μ = 0.1, k = 10	-23.878
p = 0.75, μ = 0.2, k = 1	-18.735
p = 0.75, μ = 0.2, k = 2	-34.163
p = 0.75, μ = 0.2, k = 5	36.694
p = 0.75, μ = 0.2, k = 10	44.776
p = 0.75, μ = 0.3, k = 1	-21.837
p = 0.75, μ = 0.3, k = 2	-13.224
p = 0.75, μ = 0.3, k = 5	40.531
p = 0.75, μ = 0.3, k = 10	79.02
p = 0.95, μ = 0.1, k = 15	437.388
p = 0.95, μ = 0.2, k = 15	389.184
p = 0.95, μ = 0.3, k = 15	352.531
p = 0.85, μ = 0.1, k = 15	221.265
p = 0.85, μ = 0.2, k = 15	246.327
p = 0.85, μ = 0.3, k = 15	106.163
p = 0.75, μ = 0.1, k = 15	150.041
p = 0.75, μ = 0.2, k = 15	83.755
p = 0.75, μ = 0.3, k = 15	59.102

Results for Edge-Correlated T-SBM Networks	
Dataset Name	Auto Correlation Score
p = 0.8, μ = 0.1, k = 1	6378.857
p = 0.8, μ = 0.1, k = 2	6953.755
p = 0.8, μ = 0.1, k = 5	6662.49
p = 0.8, μ = 0.1, k = 10	6945.796
p = 0.8, μ = 0.2, k = 1	6470.204
p = 0.8, μ = 0.2, k = 2	7184.776
p = 0.8, μ = 0.2, k = 5	7960.0
p = 0.8, μ = 0.2, k = 10	7052.571
p = 0.8, μ = 0.3, k = 1	6509.02
p = 0.8, μ = 0.3, k = 2	6776.735
p = 0.8, μ = 0.3, k = 5	7589.714
p = 0.8, μ = 0.3, k = 10	7799.02
p = 0.7, μ = 0.1, k = 1	4789.837
p = 0.7, μ = 0.1, k = 2	6205.714
p = 0.7, μ = 0.1, k = 5	7567.306
p = 0.7, μ = 0.1, k = 10	6521.388
p = 0.7, μ = 0.2, k = 1	4580.0
p = 0.7, μ = 0.2, k = 2	5473.265
p = 0.7, μ = 0.2, k = 5	5338.694
p = 0.7, μ = 0.2, k = 10	6118.571
p = 0.7, μ = 0.3, k = 1	4450.082
p = 0.7, μ = 0.3, k = 2	5457.469
p = 0.7, μ = 0.3, k = 5	5289.306
p = 0.7, μ = 0.3, k = 10	6347.061
p = 0.6, μ = 0.1, k = 1	3559.347
p = 0.6, μ = 0.1, k = 2	4091.02
p = 0.6, μ = 0.1, k = 5	5165.918
p = 0.6, μ = 0.1, k = 10	5894.245
p = 0.6, μ = 0.2, k = 1	3486.816
p = 0.6, μ = 0.2, k = 2	4024.367
p = 0.6, μ = 0.2, k = 5	5539.061
p = 0.6, μ = 0.2, k = 10	4957.918
p = 0.6, μ = 0.3, k = 1	3987.796
p = 0.6, μ = 0.3, k = 2	3872.816
p = 0.6, μ = 0.3, k = 5	3484.939
p = 0.6, μ = 0.3, k = 10	4593.796
p = 0.8, μ = 0.1, k = 15	6278.694
p = 0.8, μ = 0.2, k = 15	6585.224
p = 0.8, μ = 0.3, k = 15	6380.612
p = 0.7, μ = 0.1, k = 15	6259.469
p = 0.7, μ = 0.2, k = 15	6304.449
p = 0.7, μ = 0.3, k = 15	4847.878
p = 0.6, μ = 0.1, k = 15	4896.776
p = 0.6, μ = 0.2, k = 15	5405.469
p = 0.6, μ = 0.3, k = 15	4526.204

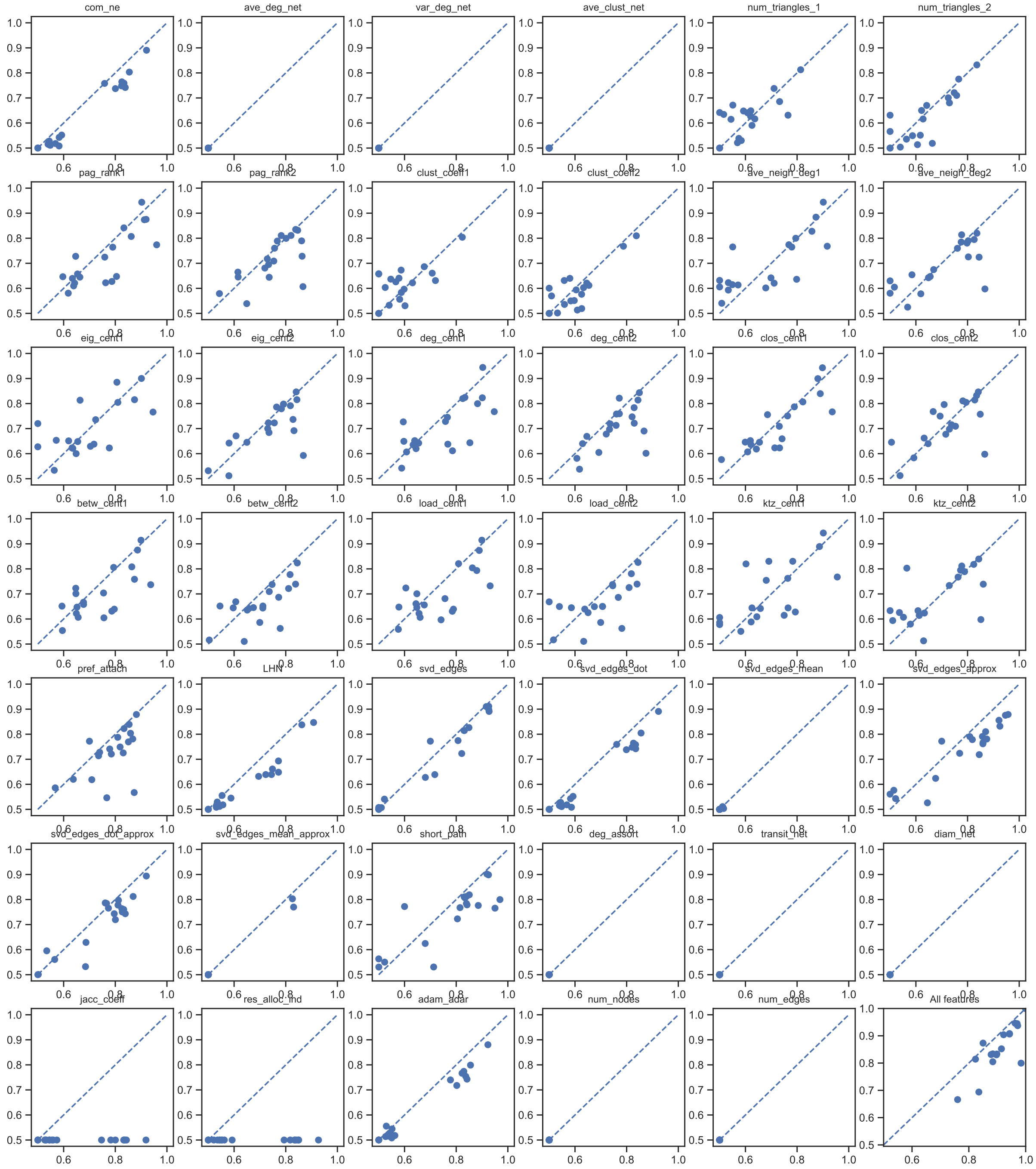

Figure SI1: The AUC plot for each features for time series and temporal topological features. Real-world Networks.

F: Time-Series Scores

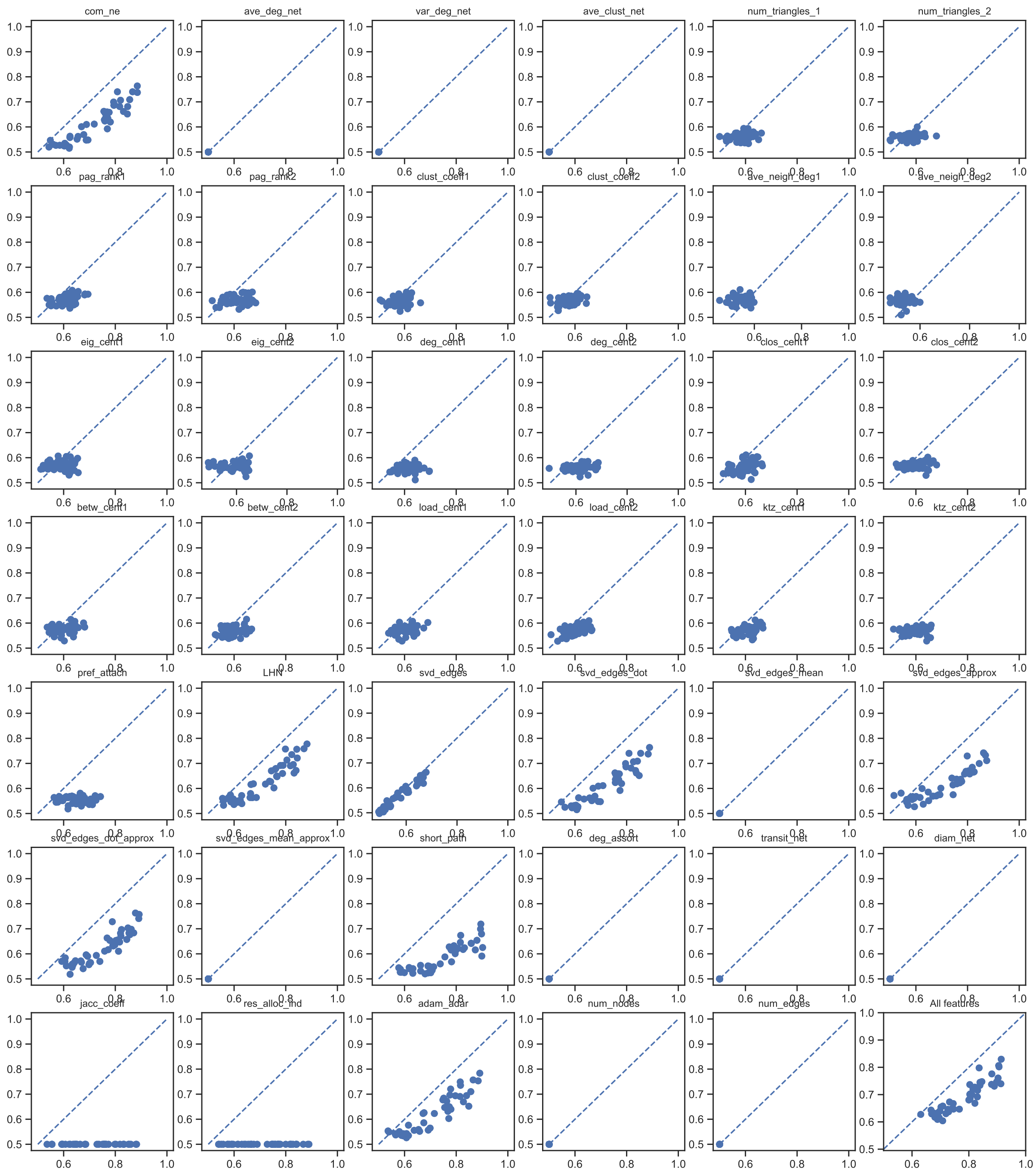

Figure SI2: The AUC plot for each features for time series and temporal topological features. Community-label T-SBMs.

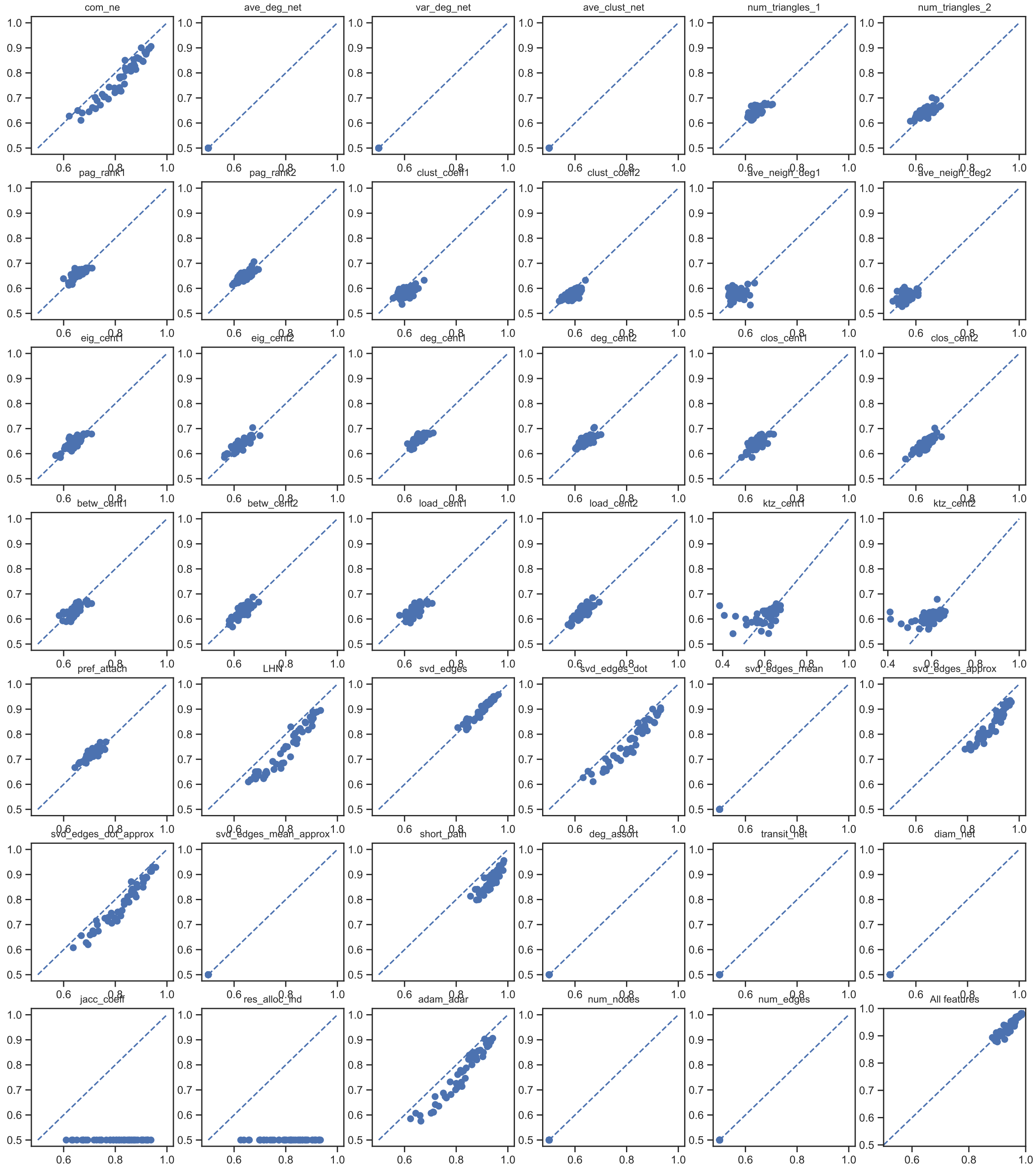

Figure SI3: The AUC plot for each features for time series and temporal topological features. Edge-Correlated T-SBMs.

Supplementary References

1. A. Clauset, E. Tucker, M. Sainz, ”The Colorado Index of Complex Networks .”, `https://icon.colorado.edu/` (2016).

2. J. Leskovec, A. Krevl, SNAP Datasets: Stanford Large Network Dataset Collection, `http://snap.stanford.edu/data` (2014).

3. R. Rossi, N. Ahmed, *Twenty-Ninth AAAI Conference on Artificial Intelligence* (2015).

4. P. Panzarasa, T. Opsahl, K. M. Carley, *Journal of the American Society for Information Science and Technology* **60**, 911 (2009).

5. S. Kumar, F. Spezzano, V. Subrahmanian, C. Faloutsos, *Data Mining (ICDM), 2016 IEEE 16th International Conference on* (IEEE, 2016), pp. 221–230.

6. S. Kumar, *et al.*, *Proceedings of the Eleventh ACM International Conference on Web Search and Data Mining* (ACM, 2018), pp. 333–341.

7. J. Kunegis, *Proceedings of the 22nd International Conference on World Wide Web* (2013), pp. 1343–1350.

8. L. E. Rocha, F. Liljeros, P. Holme, *PLOS Computational Biology* **7**, e1001109 (2011).

9. R. Michalski, S. Palus, P. Kazienko, *International Conference on Business Information Systems* (Springer, 2011), pp. 197–206.

10. N. Eagle, A. S. Pentland, *Personal and Ubiquitous Computing* **10**, 255 (2006).

11. F. Munoz-Mendez, K. Han, K. Klemmer, S. Jarvis, *Proceedings of the 2018 ACM International Joint Conference and 2018 International Symposium on Pervasive and Ubiquitous Computing and Wearable Computers* (2018), pp. 1015–1023.

12. D. P. Mersch, A. Crespi, L. Keller, *Science* **340**, 1090 (2013).

13. K. Newaz, T. Milenkovic, *IEEE/ACM Transactions on Computational Biology and Bioinformatics* (2020).

14. M. Newman, *Networks* (Oxford University Press, 2018).

15. D. Liben-Nowell, J. Kleinberg, *Journal of the American Society for Information Science and Technology* **58**, 1019 (2007).

16. E. A. Leicht, P. Holme, M. E. Newman, *Physical Review E* **73**, 026120 (2006).

17. A. Hagberg, P. Swart, D. S Chult, Exploring Network Structure, Dynamics, and Function using NetworkX, *Tech. rep.*, Los Alamos National Lab.(LANL), Los Alamos, NM (United States) (2008).

18. W. Cukierski, B. Hamner, B. Yang, *The 2011 International Joint Conference on Neural Networks* (IEEE, 2011), pp. 1237–1244.

19. A. Ghasemian, P. Zhang, A. Clauset, C. Moore, L. Peel, *Physical Review X* **6**, 031005 (2016).

20. A. Ghasemian, H. Hosseinmardi, A. Galstyan, E. M. Airoldi, A. Clauset, *Proceedings of the National Academy of Sciences* **117**, 23393 (2020).

21. L. G. S. Jeub, M. Bazzi, "A generative model for mesoscale structure in multilayer networks implemented in MATLAB,", `https://github.com/MultilayerBenchmark/MultilayerBenchmark/` (2019) version 2.0. Accessed: 2020-1-10.

22. A. R. Pamfil, S. D. Howison, M. A. Porter, *Physical Review E* **102**, 062307 (2020).

23. M. Tarrés-Deulofeu, A. Godoy-Lorite, R. Guimera, M. Sales-Pardo, *Physical Review E* **99**, 032307 (2019).

24. J. Chen, *et al.*, *IEEE Transactions on Systems, Man, and Cybernetics: Systems* **51**, 3699 (2021).
